# Supplementary material for: Prospecting Biomarkers for Diagnostic and Therapeutic Approaches in Pythiosis
Source: J Fungi (Basel). 2021 May 28;7(6):423. doi: 10.3390/jof7060423 (PMC8229905; doi:10.3390/jof7060423)
Supplement: Supplementary file 1 [file jof-07-00423-s001.zip › Supplemental materials/Supplemental material.pdf]

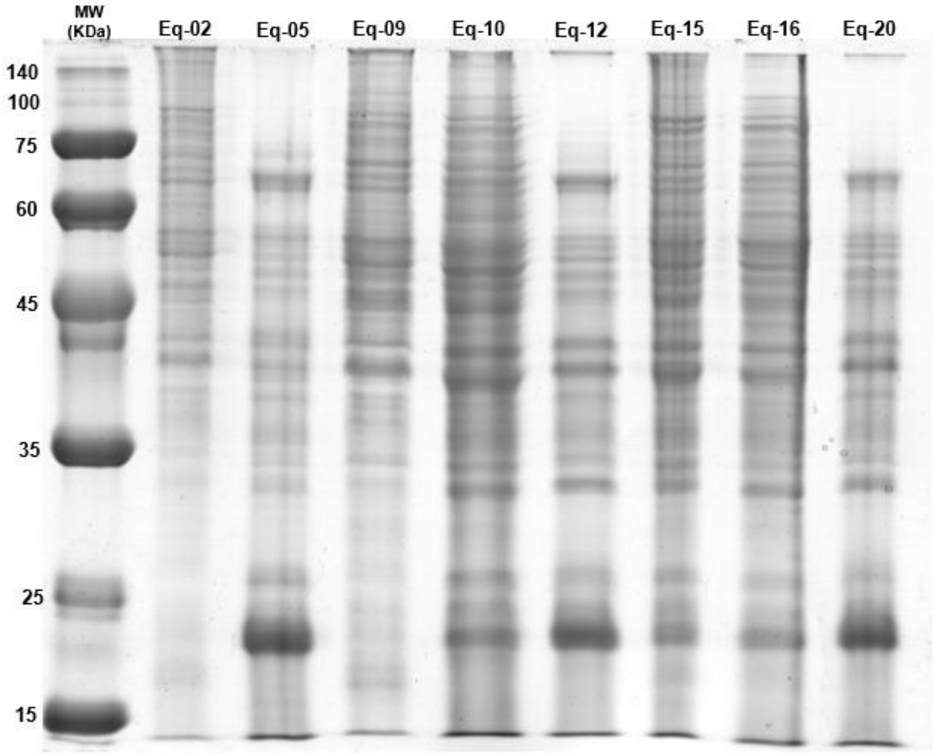

Fig S1 - The protein profiles of the eight different *Pythium insidiosum* isolates show that are similar in terms of the number and molecular weight of bands observed in the unidimensional electrophoresis.

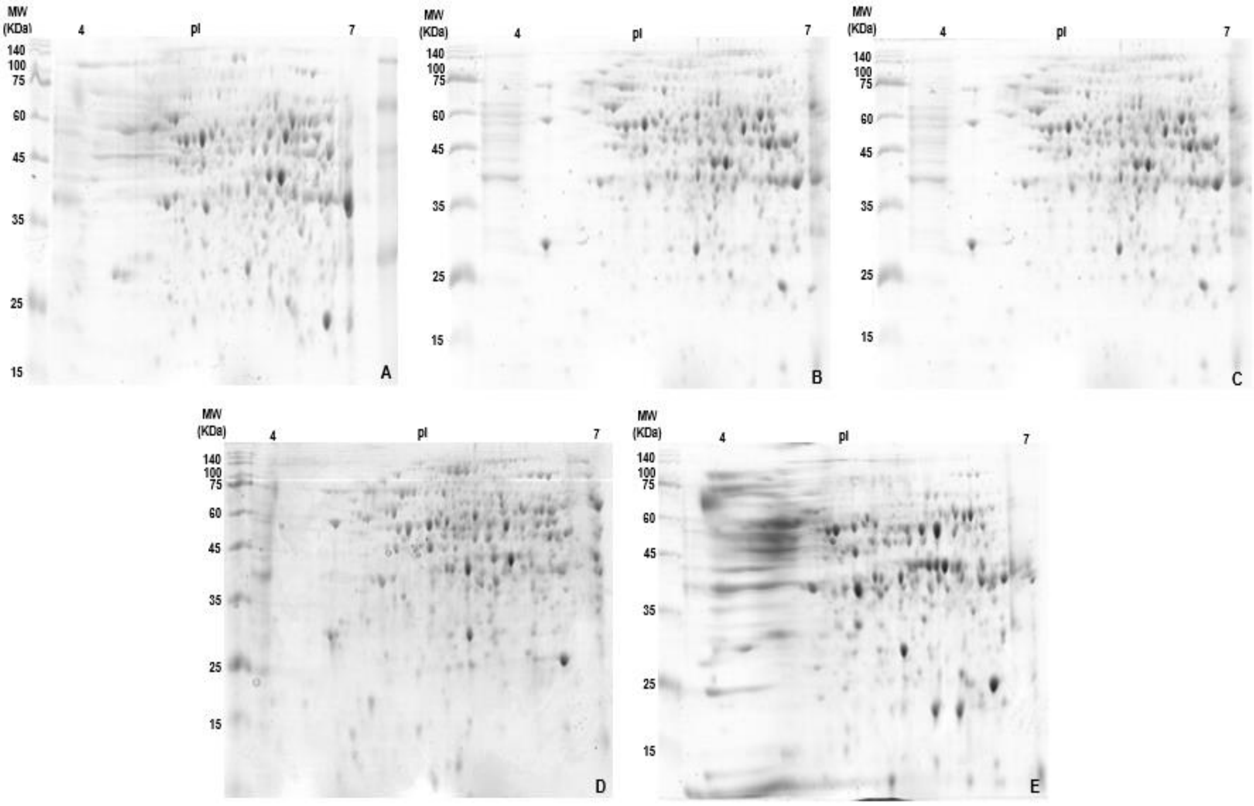

Fig S2 - *Pythium insidiosum* proteins fractionated using strips of 7cm and pH 4-7, in the first dimension, and 12.5% polyacrylamide gels in the second dimension. (A) *Pythium insidiosum* denominated “Eq-10”. (B) "Eq-12". (C) "Eq-15". (D) "Eq-16". (E) "Eq-20".

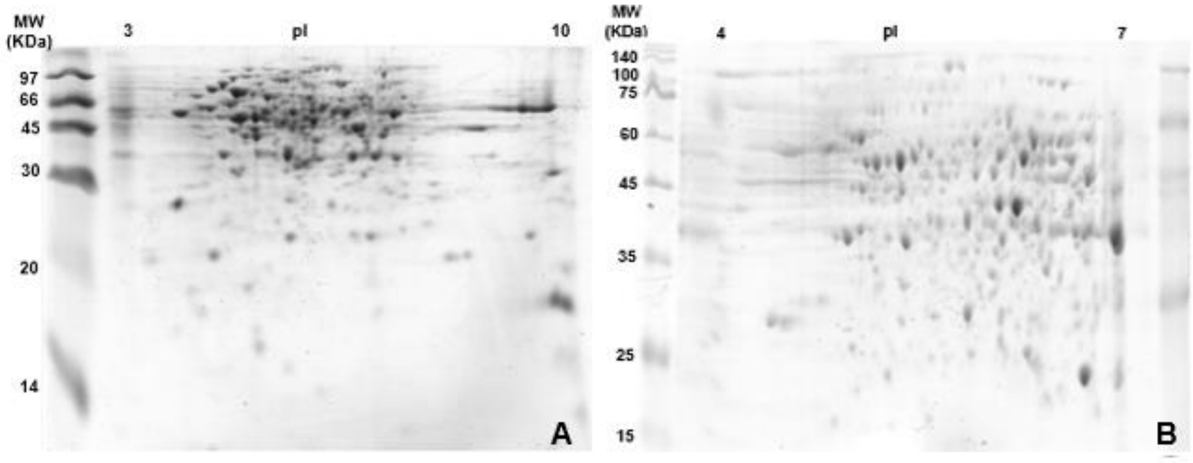

Fig S3 - Proteins (150 µg) of *Pythium insidiosum* (Eq-10) resolved by two-dimensional gel electrophoresis. (A) IPG strips ranging from 3 to 10. (B) Separation of spots by isoelectric point using strips with a pH range of 4-7.
